# Simplifying surgical criteria for early-stage cervical cancer: prognostic equivalence of total vs. radical hysterectomy in a retrospective SEER cohort analysis

**DOI:** 10.1097/JS9.0000000000002731

**Published:** 2025-06-13

**Authors:** Yifan Meng, Lanyue Cui, Cheng Fang, Fan Yang, Jundong Li, Ting Wan

**Affiliations:** Department of Gynecologic Oncology, State Key Laboratory of Oncology in South China, Collaborative Innovation Center for Cancer Medicine, Sun Yat-sen University Cancer Center, Guangzhou, Guangdong, China

**Keywords:** conservative hysterectomy, early-stage cervical cancer, gynecologic oncology, prognosis, retrospective cohort study, SEER, surgery, survival, United States

## Abstract

**Background:**

The prognostic equivalence of total hysterectomy (TH) versus radical hysterectomy (RH) in early-stage cervical cancer (IA2-IB1) with tumor size ≤2 cm remains controversial, particularly regarding the necessity of lymphovascular space invasion (LVSI) assessment. This study evaluates survival outcomes under simplified criteria omitting LVSI and depth of invasion evaluation.

**Materials and methods:**

This retrospective cohort study analyzed 3002 FIGO IA2-IB1 cervical cancer patients (tumors ≤2 cm) from the SEER database (2004–2019). Inclusion criteria are histologically confirmed adenocarcinoma, adenosquamous carcinoma, or squamous cell carcinoma; TH/RH with lymphadenectomy/sentinel node biopsy. Outcomes included overall survival (OS) and disease-specific survival (DSS), analyzed via Kaplan–Meier, Cox regression, and propensity score matching (PSM).

**Results:**

Median follow-up was 73 months. No significant differences were observed in OS (92.3% vs. 92.3%, *P* = 0.74) and DSS (96.4% vs. 96.6%, *P* = 0.89) outcomes between RH and TH cohorts, consistent across FIGO stages and adjuvant therapy-without patients. Multivariable analysis confirmed age >49 years (HR = 2.50, 95% CI = 1.91–3.28, *P* < 0.01), marital status of separated/divorced/widowed (HR = 1.66, 95% CI = 1.20–2.28, *P* < 0.01), and tumor size 11–20 mm (HR = 1.61, 95% CI = 1.18–2.19, *P* < 0.01) as independent risk factors in OS. While surgical approach still showed no prognostic significance both in OS (HR = 1.04, 95% CI = 0.79–1.37, *P* = 0.77) and DSS (HR = 1.01, 95% CI = 0.67–1.53, *P* = 0.96). Post-PSM analysis (*n* = 2,715) confirmed survival equivalence (*P* > 0.05). However, in IB1 adenosquamous/adenocarcinoma patients aged >49 years with tumors 11–20 mm, RH achieved superior DSS (*P* = 0.01), though OS differences were nonsignificant (*P* = 0.085). Squamous carcinoma outcomes remained equivalent regardless of surgery (*P* = 0.43).

**Conclusion:**

TH achieves survival outcomes comparable to RH in most early-stage cervical cancer patients with tumors ≤2 cm, supporting its application in low-risk populations. However, RH remains preferred for stage IB1 patients with adenocarcinoma or adenosquamous carcinoma aged >49 years and tumors measuring 11–20 mm. Simplified criteria omitting LVSI and stromal depth assessment may enhance accessibility in resource-limited settings without compromising safety.

## Introduction

Cervical cancer remains the most common gynecologic malignancy and the fourth leading cause of cancer-related mortality among women globally^[1]^. It continues to pose a significant public health burden, with disproportionate impacts on populations in resource-limited settings. While Scandinavian nations maintain low incidence (8–10/100 000) through routine HPV vaccination and population-wide screening, sub-Saharan Africa faces rates over 40/100 000^[2]^, which was driven by limited screening access, weak health systems, and high HIV co-infection. This stark contrast highlights the urgent need for clinical guidelines tailored to local resource realities^[3]^.

For early-stage cervical cancer (stages IA2 to IB1), radical hysterectomy (RH) with pelvic lymph node dissection has long been the standard surgical approach, offering excellent oncologic outcomes^[4-6]^. However, RH is associated with significant short- and long-term complications, including urinary, bowel, and sexual dysfunction, which can profoundly impact patients’ quality of life (QoL)^[7-12]^. In recent years, there has been growing interest in conservative surgical approaches, such as total hysterectomy (TH), for low-risk early-stage cervical cancer. The ConCerv study proposed six criteria for selecting patients suitable for TH, including having negative margins, negative lymphovascular space invasion (LVSI), squamous cell (any grade) or usual type adenocarcinoma (grade 1 or 2 only), tumor size ≤2 cm, depth of invasion ≤10 mm, and negative imaging for metastatic disease^[13-15]^. Based on the ConCerv study, the National Comprehensive Cancer Network (NCCN) guidelines updated their recommendations in 2023, acknowledging TH as a viable alternative for low-risk patients^[16,17]^. The recently published SHAPE study also demonstrated that TH was non-inferior to RH in terms of 3-year pelvic recurrence rates (2.52% vs. 2.17%, *P* = 0.35) and was associated with fewer complications, further supporting the safety of conservative surgery^[14]^.

Despite these advancements, critical challenges persist in surgical triage criteria. Inconsistent inclusion criteria for conservative surgery eligibility remain across major studies, exemplified by the SHAPE trial’s exclusion of adenocarcinoma differentiation requirements compared to ConCerv’s histological emphasis. The inclusion of LVSI remains contentious among studies: the ConCerv trial mandated LVSI negativity, whereas the SHAPE trial imposed no such restriction. Furthermore, the evaluation of patients without preoperative LEEP (loop electrosurgical excision procedure) or cone biopsy warrants further investigation. These compounded limitations collectively underscore the urgent need for simplified, standardized criteria that reconcile preoperative assessment reliability with surgical decision-making pragmatism.

In this context, our study aims to address these gaps by evaluating the prognostic outcomes of TH versus RH in a large, population-based cohort from the SEER database. Specifically, we focus on stage IB1 cervical cancer patients with tumor sizes ≤2 cm, excluding the requirement for LVSI and depth of invasion assessment. By doing so, we aim to identify a more streamlined and clinically practical set of criteria for selecting patients suitable for conservative surgery. This retrospective cohort study has been reported in line with the STROCSS 2025 guidelines^[18]^.

## Materials and methods

### Population registries

**Figure 1.**
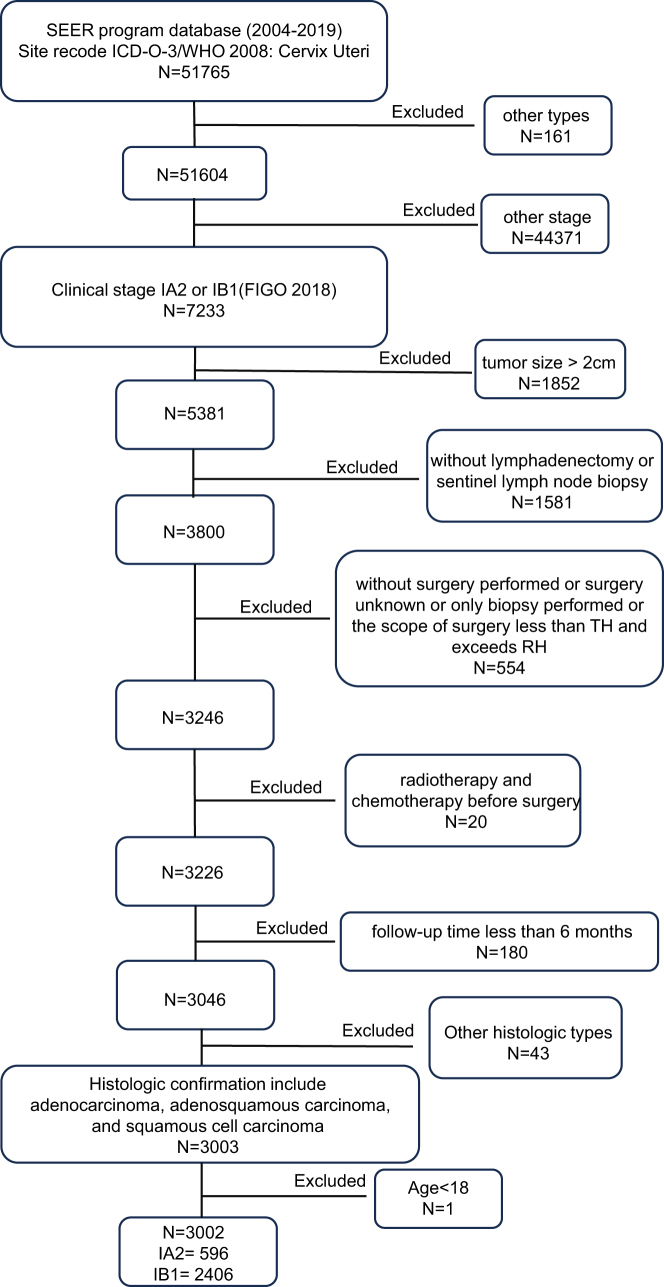
Flowchart of patient selection criteria for early-stage cervical cancer patients. Histologic subtypes were classified using ICD-O-3 codes as implemented by the SEER program: adenocarcinoma (8140, 8144, 8263, etc.), adenosquamous carcinoma (8560, 8570, and 8015), and squamous cell carcinoma (8051–8052, 8070–8076, 8082–8083). For code definitions, refer to WHO ICD-O-3 guidelines (2000) and SEER coding documentation.

The data on cervical cancer patients diagnosed between 2004 and 2019 were acquired using the SEER*Stat software, version 8.4.5, from the Surveillance, Epidemiology, and End Results (SEER) Research Plus Data of 17 Registries. FIGO 2018 staging was reconstructed using AJCC 6th edition TNM staging and SEER Combined Stage Group, with tumor size >2 cm as a key exclusion criterion. Inclusion criteria: (1) histologically confirmed cervical adenocarcinoma, adenosquamous carcinoma, or squamous cell carcinoma; (2) clinical stage IA2 or IB1; (3) underwent TH or RH with/without salpingo-oophorectomy; (4) age>18 years old; (5) tumor size ≤:2cm. Exclusion criteria: (1) no lymphadenectomy/sentinel lymph node biopsy; (2) no surgery, unknown surgery, biopsy-only, or surgical scope less than TH but exceeding RH; (3) preoperative chemotherapy/radiotherapy; (4) follow-up <6 months. (Supplementary Digital Content 1, Table S1, available at: http://links.lww.com/JS9/E359) summarizes the inclusion and exclusion criteria, and Figure 1 illustrates the flow chart of participant inclusion and exclusion criteria. Depth of invasion assessment was omitted due to inconsistent requirements in pivotal trials and limited preoperative feasibility: accurate measurement requires cone biopsy specimens, while MRI interpretation shows suboptimal correlation with final pathology. A total of 3,002 patients with early-stage cervical cancer (FIGO IA2–IB1) were included, comprising 2,009 radical hysterectomy (RH) and 993 total hysterectomy (TH) cases. The institutional review board exempted this study from review and informed consent due to the use of deidentified data. Follow-up was conducted by the SEER registries through active patient tracking, including linkage to vital status records. No incentives were provided, as data were de-identified and retrospective.

**HIGHLIGHTS**

- TH demonstrates comparable long-term survival to RH in low-risk early-stage cervical cancer (tumor size ≤2 cm, FIGO2018 stage IA1-IB1), without requiring LVSI or stromal invasion depth assessment.
- Using tumor size and FIGO stage alone streamlines surgical eligibility criteria, reducing reliance on complex histopathologic variables (e.g., LVSI) while preserving oncologic safety, potentially expanding access to less invasive procedures.
- RH remains preferred for stage IB1 adenosquamous/adenocarcinoma patients aged >49 years with tumors 11–20 mm due to their inherently poorer prognosis, necessitating more aggressive surgical intervention despite early-stage classification.

### Study variables

Clinicopathological variables for collected data in this analysis included: surgery, race, year of diagnosis, age, marital status, stage, grade, histology, stage, tumor size, postoperative radiotherapy, chemotherapy, and survival months. According to the surgical procedures recorded in SEER data, we have categorized patients into two groups, namely TH (patients who underwent total hysterectomy), and RH (patients who underwent radical hysterectomy). The stage of included patients was assessed by FIGO stage based on the American Joint Committee on Cancer (AJCC) 6th, TNM stage and SEER Combined Stage Group^[19]^. The primary endpoint of the study was overall survival (OS), and the secondary endpoint of the study was disease-specific survival (DSS).

## Statistical analysis

The baseline categorical characteristics were compared with χ^2^ test or Fisher’s exact test. Kaplan-Meier survival curves and log-rank tests were employed for univariate analysis. Propensity score matching (PSM) with a 1:2 nearest-neighbor algorithm (caliper = 0.05) was performed using age, histologic grade, histology, FIGO stage, diagnosis year, marital status, and tumor size. The Cox proportional-hazards model was used for multivariate survival analyses. OS was the primary outcome, and DSS was the secondary outcome. Subgroup analyses were predefined for patients with stage IB1, with particular focus on histological, age-related, and tumor size-related subtypes, which exhibited discrepancies in multivariate Cox regression analysis. Survival comparisons were stratified by these clinicopathological features to identify potential effect modifiers. Two-sided *P* < 0.05 was considered to be significant. This study followed the STROBE reporting guideline. All analyses were performed using R statistical software (version 4.4.3) and IBM SPSS Statistics 27. Artificial intelligence was not utilized in any stage of this research or manuscript development.

## Results

A total of 3002 patients with early-stage cervical cancer (FIGO IA2–IB1) were included, comprising 2009 RH and 993 TH cases. The median follow-up time was 73 months (interquartile range: 35–125 months), allowing us to effectively assess the favorable prognosis of low-risk early-stage cervical cancer. Baseline characteristics revealed significant intergroup disparities in year of diagnosis (*P* < 0.01) and tumor grade (*P* = 0.04), with TH patients exhibiting higher proportions of grade I tumors (RH 15.06% vs. TH 18.50%) (Table 1).

**Table 1:** **Baseline clinicopathologic characteristics of radical hysterectomy** **(RH) and total hysterectomy (TH) cohorts**

Characteristics	Overall (*n* = 3002)	RH (*n* = 2009)	TH (*n* = 993)	*P*
Race (%)				
Black	263 (8.86)	183 (9.23)	80 (8.11)	*0.13*
White	2345 (79.01)	1574 (79.41)	771 (78.19)	
Other	360 (12.13)	225 (11.35)	135 (13.69)	
Year of diagnosis (%)				
2004–2009	1037 (34.54)	727 (36.19)	310 (31.22)	*<0.01*
2010–2014	897 (29.88)	559 (27.82)	338 (34.04)	
2015–2019	1068 (35.58)	723 (35.99)	345 (34.74)	
Age (%)				
19–49	2067 (68.85)	1401 (69.74)	666 (67.07)	*0.15*
>49	935 (31.15)	608 (30.26)	327 (32.93)	
Marital status (%)				
Married/Partnered	1544 (53.76)	1029 (53.32)	515 (54.67)	*0.06*
Single	830 (28.90)	582 (30.16)	248 (26.33)	
Separated/Divorced/Widowed	498 (17.34)	319 (16.53)	179 (19.00)	
Grade (%)				
Grade1	425 (16.19)	265 (15.06)	160 (18.50)	*0.04*
Grade2	1344 (51.20)	901 (51.19)	443 (51.21)	
Grade3	856 (32.61)	594 (33.75)	262 (30.29)	
Histology (%)				
Adenocarcinoma	816 (27.18)	534 (26.58)	282 (28.40)	*0.52*
Adenosquamous carcinoma	140 (4.66)	92 (4.58)	48 (4.83)	
Squamous cell carcinoma	2046 (68.15)	1383 (68.84)	663 (66.77)	
Stage (%)				
IA2	596 (19.85)	393 (19.56)	203 (20.44)	*0.60*
IB1	2406 (80.15)	1616 (80.44)	790 (79.56)	
Tumor size (%)				
≤10 mm	1084 (46.48)	726 (46.66)	358 (46.13)	*0.85*
11–20 mm	1248 (53.52)	830 (53.34)	418 (53.87)	
Radiotherapy (%)				
None/Unknown	2630 (87.61)	1762 (87.71)	868 (87.41)	*0.86*
Yes	372 (12.39)	247 (12.29)	125 (12.59)	
Chemotherapy (%)				
No/Unknown	2813 (93.70)	1880 (93.58)	933 (93.96)	*0.75*
Yes	189 (6.30)	129 (6.42)	60 (6.04)	
OS (%)				
Alive	2771 (92.31)	1855 (92.33)	916 (92.25)	*0.99*
Dead	231 (7.69)	154 (7.67)	77 (7.75)	
DSS (%)				
Alive	2896 (96.47)	1937 (96.42)	959 (96.58)	*0.91*
Dead	106 (3.53)	72 (3.58)	34 (3.42)	
				
Time (median [IQR])	73.00 [35.00, 125.00]	74.00 [35.00, 128.00]	72.00 [33.00, 119.00]	*0.30*

DSS, disease-specific survival; OS, overall survival; RH, radical hysterectomy; TH, total hysterectomy.Categorical variables analyzed using χ^2^ tests or Fisher’s exact test; continuous variable (Time) analyzed using Mann-Whitney U test.

**Figure 2.**
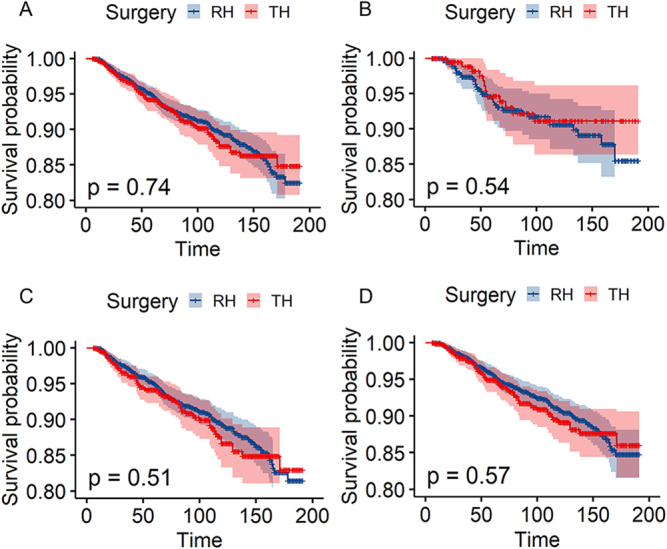
Kaplan–Meier survival analysis of overall survival in early-stage cervical cancer: radical hysterectomy(RH) versus total hysterectomy(TH) (A) entire cohort; (B) stage IA2 subgroup; (C) stage IB1 subgroup; (D) patients without adjuvant therapy subgroup.

Kaplan–Meier analysis demonstrated comparable OS and DSS outcomes between RH and TH cohorts (OS: 92.3% vs. 92.3%, *P* = 0.74; Figure 2A; DSS: 96.4% vs. 96.6%, *P* = 0.89; (Supplementary Digital Content 1, Fig. S1A, available at: http://links.lww.com/JS9/E359). This equivalence persisted across FIGO stage subgroups, as evidenced by OS (*P* = 0.54; Figure 2B) and DSS (*P* = 0.68; Supplementary Digital Content 1, Fig. S1B, available at: http://links.lww.com/JS9/E359) in stage IA2 patients, while stage IB1 patients showed similar OS (*P* = 0.51; Figure 2C) and DSS rates (*P* = 0.80; Supplementary Digital Content 1, Fig. S1C, available at: http://links.lww.com/JS9/E359). Notably, among adjuvant therapy-naive patients (*n* = 2600), survival outcomes remained statistically indistinguishable between surgical approaches, with RH demonstrating OS versus TH (*P* = 0.57; Figure 2D) and DSS (*P* = 0.86; Supplementary Digital Content 1, Fig. S1D, available at: http://links.lww.com/JS9/E359). Five-year survival analysis (Supplementary Digital Content 1, Table S2, available at: http://links.lww.com/JS9/E359) further confirmed these findings: RH and TH exhibited nearly identical OS and DSS rates in the overall cohort, with no significant differences in stage-stratified subgroups or among patients without adjuvant therapy. These multi-dimensional analyses robustly support the equivalence of long-term survival outcomes between surgical approaches.Univariate Cox analysis (Table 2) identified year of diagnosis (2015–2019: HR = 1.58, 95% CI = 1.02–2.45, *P* = 0.04), age >49 years (HR = 2.89, 95% CI = 2.23–3.74, *P* < 0.01), marital status of Separated/Divorced/Widowed (HR = 2.16, 95% CI = 1.58–2.94, *P* < 0.01), grade (II: HR = 1.69, 95% CI = 1.05–2.73, *P* = 0.03; III: HR = 2.10, 95% CI = 1.29–3.43, *P* < 0.01), and tumor size 11–20 mm (HR = 1.87, 95% CI = 1.39–2.52, *P* < 0.01) as the mortality predictors in OS, while surgical approach showed no prognostic significance (OS: HR = 1.05, 95% CI = 0.80–1.38, *P* = 0.74; DSS: HR = 0.97, 95% CI = 0.65–1.46, *P* = 0.89). Additionally, histology (adenosquamous carcinoma: HR = 2.37, 95% CI = 1.20–4.66, *P* = 0.01) and stage (IB1: HR = 2.65, 95% CI = 1.38–5.09, *P* < 0.01) were further considered as the mortality predictors in DSS. The cohort of patients receiving adjuvant therapy had poor OS (radiotherapy: HR = 2.22, 95% CI = 1.59–3.10, *P* < 0.01; chemotherapy: HR = 2.42, 95% CI = 1.56–3.76, *P* < 0.01) and DSS (radiotherapy: HR = 3.13, 95% CI = 2.01–4.87, *P* < 0.01; chemotherapy: HR = 4.20, 95% CI = 2.50–7.07, *P* < 0.01).

**Table 2:** Univariate Cox regression analysis for OS and DSS in stage IA2-IB1 patients before PSM

Characteristics	OS			DSS		
	HR	CI95	*P*	HR	CI95	*P*
Race						
Black	1			1		
White	0.97	0.62–1.51	*0.90*	0.76	0.41–1.39	*0.38*
Other	0.88	0.49–1.56	*0.66*	0.84	0.39–1.85	*0.67*
Year of diagnosis						
2004–2009	1			1		
2010–2014	1.02	0.74–1.40	*0.93*	1.32	0.80–1.97	*0.32*
2015–2019	1.58	1.02–2.45	*0.04*	1.88	1.06–3.34	*0.03*
Age						
19–49	1					
>49	2.89	2.23–3.74	*<0.01*	1.49	1.01–2.21	*0.05*
Marital status						
Married/Partnered	1			1		
Separated/Divorced/Widowed	2.16	1.58–2.94	*<0.01*	1.07	0.41–2.81	*0.89*
Single	1.30	0.94–1.79	*0.11*	0.82	0.32–2.14	*0.69*
Grade						
Grade I	1			1		
Grade II	1.69	1.05–2.73	*0.03*	3.46	1.38–8.66	*0.01*
Grade III	2.10	1.29–3.43	*<0.01*	4.20	1.66–10.65	*<0.01*
Histology						
Adenocarcinoma	1					
Adenosquamous carcinoma	1.78	1.01–3.12	*0.05*	2.37	1.20–4.66	*0.01*
Squamous cell carcinoma	1.34	0.97–1.84	*0.07*	0.95	0.61–1.49	*0.89*
Stage						
IA2	1			1		
IB1	1.28	0.92–1.79	*0.14*	2.65	1.38–5.09	*<0.01*
Tumor size						
≤10 mm	1					
11–20 mm	1.87	1.39–2.52	*<0.01*	4.03	2.38–6.82	*<0.01*
Surgery						
RH	1			1		
TH	1.05	0.80–1.38	*0.74*	0.97	0.65–1.46	*0.89*
Radiotherapy						
None/Unknown	1			1		
Yes	2.22	1.59–3.10	*<0.01*	3.13	2.01–4.87	*<0.01*
Chemotherapy						
No/Unknown	1			1		
Yes	2.42	1.56–3.76	*<0.01*	4.20	2.50–7.07	*<0.01*

HR, hazard ratio. Analyses used univariate Cox proportional hazards regression.

The variables that showed significance (*P* < 0.05) in univariate Cox regression analysis and surgery were subjected to the multivariate Cox regression analysis. Multivariable analysis (Table 3) confirmed age >49 (HR = 2.50, 95% CI = 1.91–3.28, *P* < 0.01), marital status of Separated/Divorced/Widowed (HR = 1.66, 95% CI = 1.20–2.28, *P* < 0.01), and tumor size 11–20 mm (HR = 1.61, 95% CI = 1.18–2.19, *P* < 0.01) as independent risk factors in OS, grade (II: HR = 3.43, 95% CI = 1.34–8.82, *P* = 0.01; III: HR = 3.73, 95% CI = 1.41–9.91, *P* = 0.01) were further considered as the independent risk factors in DSS. While surgical approach still showed no prognostic significance both in OS (HR = 1.04, 95% CI = 0.79–1.37, *P* = 0.77) and DSS (HR = 1.01, 95% CI = 0.67–1.53, *P* = 0.96).

**Table 3:** Multivariate Cox regression analysis for OS and DSS in stage IA2-IB1 patients

Characteristics	OS			DSS		
	HR	CI95	*P*	HR	95% CI	*P*
Year of diagnosis						
2004–2009	1			1		
2010–2014	0.99	0.72–1.37	*0.95*	1.17	0.74–1.85	*0.50*
2015–2019	1.45	0.93–2.27	*0.11*	1.69	0.93–3.06	*0.08*
Age						
19–49	1			1		
>49	2.50	1.91–3.28	*<0.01*	1.30	0.87–1.96	*0.20*
Marital status						
Married/Partnered	1			1		
Separated/Divorced/Widowed	1.66	1.20–2.28	<0.01	1.26	0.76–2.09	*0.37*
Single	1.32	0.95–1.82	*0.10*	1.03	0.64–1.65	*0.91*
Histology						
Adenocarcinoma	1			1		
Adenosquamous carcinoma	1.46	0.82–2.62	*0.20*	1.57	0.77–3.19	*0.21*
Squamous cell carcinoma	1.03	0.73–1.45	*0.87*	0.70	0.44–1.11	*0.13*
Grade						
Grade I	1			1		
Grade II	1.42	0.86–2.36	*0.17*	3.43	1.34–8.82	*0.01*
Grade III	1.57	0.93–2.66	*0.09*	3.73	1.41–9.91	*0.01*
Tumor size						
≤10 mm	1			1		
11–20 mm	1.61	1.18–2.19	*<0.01*	3.16	1.84–5.41	*<0.01*
Surgery						
RH	1			1		
TH	1.04	0.79–1.37	*0.77*	1.01	0.67–1.53	*0.96*
Radiotherapy						
None/Unknown	1			1		
Yes	1.34	0.88–2.04	*0.18*	1.54	0.85–2.78	*0.16*
Chemotherapy						
No/Unknown	1			1		
Yes	1.42	0.82–2.45	*0.22*	2.07	1.04–4.12	*0.04*

HR, hazard ratio. Analyses used multivariate Cox proportional hazards regression.

**Figure 3.**
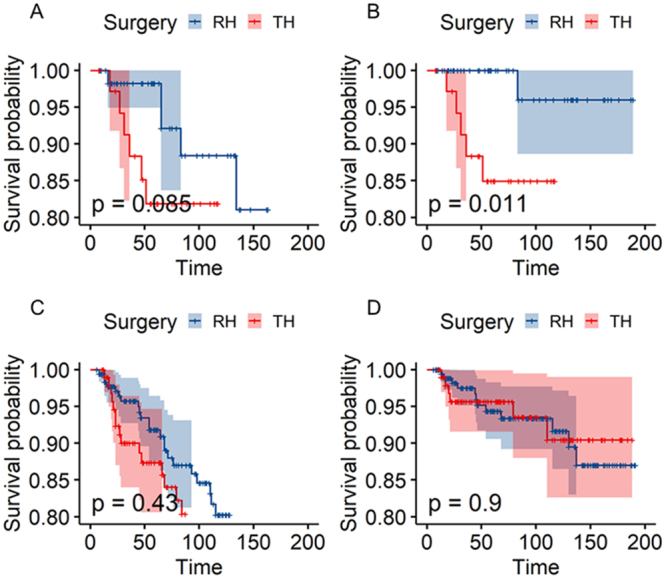
Comparison between radical and total hysterectomy cohorts after PSM at stage IB1, aged over 49 and with tumor size 11–20 mm: (A) adenocarcinoma or adenosquamous carcinoma in overall survival; (B) adenocarcinoma or adenosquamous carcinoma in disease-specific survival; (C) squamous cell carcinoma in overall survival; (D) squamous cell carcinoma in disease-specific survival.

After PSM (1:2 ratio), 2715 patients were matched, achieving balanced covariates (Supplementary Digital Content 1, Table S3 and Figure S2, available at: http://links.lww.com/JS9/E359). Post-PSM multivariable Cox regression (Supplementary Digital Content 1, Table S4, available at: http://links.lww.com/JS9/E359) confirmed no survival difference between RH and TH (OS: HR = 1.11, 95% CI = 0.84–1.48, *P* = 0.47; DSS: HR = 1.06, 95% CI = 0.70–1.63, *P* = 0.77), aligning with the Kaplan–Meier curves of the matched cohort (Supplementary Digital Content 1, Fig. S3A, available at: http://links.lww.com/JS9/E359 *P* = 0.50). Furthermore, a comparison of the surgical approach in competing risk regression analysis (Supplementary Digital Content 1, Table S6, available at: http://links.lww.com/JS9/E359, and Table S7, available at: http://links.lww.com/JS9/E359) revealed no statistically significant differences between the pre- and post-PSM cohorts (pre-PSM: Exp(coef) = 0.95, 95% CI = 0.63–1.42, *P* = 0.79; post-PSM: Exp(coef) = 0.99, 95% CI = 0.65–1.51, *P* = 0.97). Notably, even among patients without adjuvant therapy (Supplementary Digital Content 1, Fig. S3B, available at: http://links.lww.com/JS9/E359), the equivalence persisted (*P* = 0.45), reinforcing that surgical approach alone does not drive prognosis in this low-risk population. In addition, age >49 years (HR = 2.74, 95% CI = 2.04–3.67, *P* < 0.01) and tumor size 11–20 mm (HR = 1.48, 95% CI = 1.07–2.06, *P* = 0.02) were identified as independent risk factors for OS in post-PSM multivariable Cox regression (Supplementary Digital Content 1, Table S4, available at: http://links.lww.com/JS9/E359). Therefore, we further included age and tumor size as high-risk factors in stage IB1 patients for KM subgroup analysis, and subgroup survival analysis after PSM revealed nuanced prognostic patterns (Fig. 3). Among matched adenocarcinoma or adenosquamous carcinoma patients at stage IB1 with two high-risk factors (age >49 years and tumor size 11–20 mm), RH demonstrated superior DSS compared to TH (*P* = 0.01; Fig. 3B), but no statistical significance in OS (*P* = 0.085; Fig. 3A). However, squamous cell carcinoma patients at stage IB1 with two high-risk factors exhibited equivalent outcomes regardless of surgical approach (Fig. 3C–D). It is suggested that the prognostic equivalence of TH may not be applicable to adenocarcinoma or adenosquamous carcinoma patients at stage IB1 with two high-risk factors.

## Discussion

The evolution of surgical management for early-stage cervical cancer has entered a pivotal era, marked by a paradigm shift toward balancing oncologic safety with quality-of-life (QOL) preservation. Recently, several prospective multicenter studies evaluating conservative surgery in this population, as summarized in Supplementary Digital Content 1, Table S5, available at: http://links.lww.com/JS9/E359, suggest that TH may be associated with fewer intraoperative and postoperative complications compared to RH. The exploratory analyses from the SHAPE trial extension demonstrated that TH for low-risk early-stage cervical cancer offers comparable oncologic outcomes to RH while significantly improving sexual health, reducing sexual distress, and enhancing long-term quality of life^[20]^. These observations are also supported by the GOG-0278 trial, which reported that nonradical surgery appears to preserve excellent QOL with transient declines in bladder, bowel, and sexual function that largely resolve to baseline levels postoperatively^[12]^. Our population-based analysis of 3002 patients with stage IA2–IB1 cervical cancer (tumors ≤2 cm) further indicates that TH achieves long-term survival outcomes comparable to RH. These findings are consistent with landmark trials such as the SHAPE and ConCerv studies, which reported equivalent pelvic recurrence rates for TH in carefully selected low-risk cohorts^[14,17]^.

**Figure 4.**
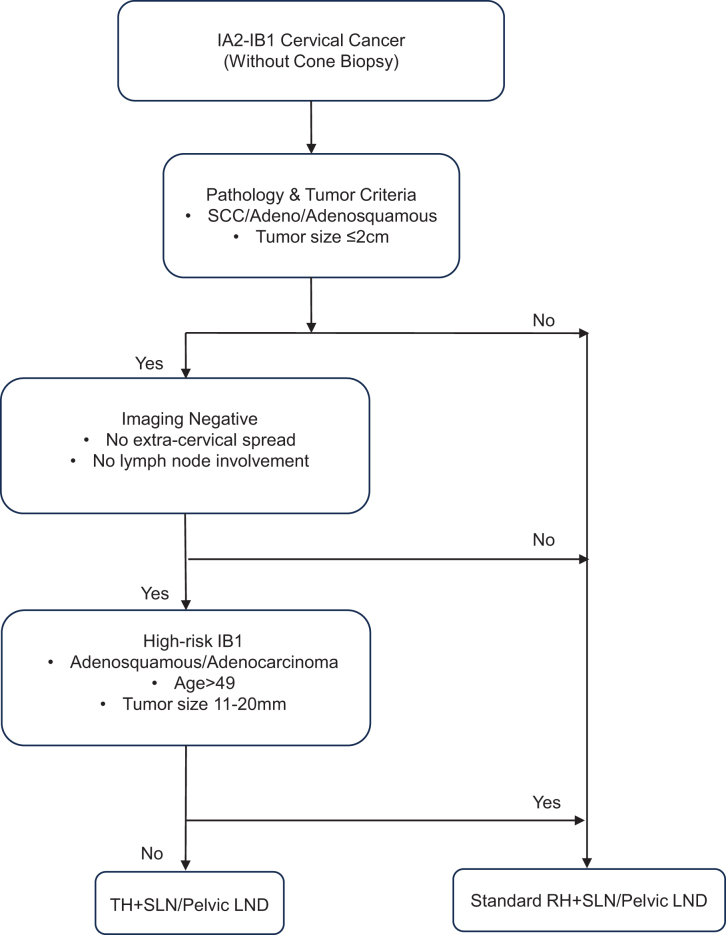
Clinical decision algorithm for conservative surgical eligibility in early-stage cervical cancer. TH, total hysterectomy; RH, radical hysterectomy; SLN, sentinel lymph node; pelvic LND, pelvic lymph node dissection.

Current guidelines (2025 NCCN v4) recommend conservative surgery for patients diagnosed via cone biopsy who meet stringent criteria, including no LVSI; negative cone margins; squamous cell (any grade) or usual type adenocarcinoma (grade 1 or 2 only); tumor size ≤2 cm; depth of invasion ≤10 mm on LEEP/cone; and negative imaging for locoregional disease (MRI recommended)^[21]^. Building on these recommendations, our study tentatively proposes simplified selection criteria for patients without preoperative cone biopsy. Specifically, TH with sentinel lymph node (SLN) mapping or pelvic lymphadenectomy might be considered for stage IA2–IB1 squamous carcinoma, adenocarcinoma, or adenosquamous carcinoma (tumors ≤2 cm) lacking radiographic evidence of extra-cervical involvement. However, for certain high-risk subgroups, such as IB1 tumors 11–20 mm in adenosquamous/adenocarcinoma patients aged >49 years, radical surgery may still be recommended. To aid clinical decision-making, we provide a practical algorithm (Figure 4) that simplifies eligibility assessment by omitting preoperative LVSI and stromal depth evaluations, which were historically emphasized but often challenging to assess preoperatively in routine practice. The grouping of adenosquamous carcinoma with adenocarcinoma for high-risk stratification was driven by two key considerations: First, while ConCerv excluded adenosquamous histology^[17]^, SHAPE included it^[14]^, and our data revealed significantly higher recurrence risk for adenosquamous carcinoma versus squamous tumors. Second, NCCN guidelines note that adenocarcinoma prognosis correlates more strongly with tumor size than depth of invasion, aligning with our decision to prioritize size-based risk stratification for glandular subtypes^[16]^.

Importantly, our findings may have implications for resource-limited regions. The omission of LVSI assessment could reduce preoperative costs and delays, particularly in regions with constrained health care infrastructure. The World Health Organization (WHO) cervical cancer guidelines emphasize the need for scalable strategies in low-income countries, where specialized histopathologic evaluation of LVSI may be unavailable. While our data do not directly quantify cost savings, simplified criteria aligning with WHO priorities could theoretically enhance access to conservative surgery without compromising safety^[22]^.

Our analysis cautiously challenges the necessity of stringent preoperative LVSI evaluation and adenocarcinoma grading. Although the ConCerv trial mandated LVSI exclusion, none of its recurrence cases exhibited LVSI positivity, raising questions about its prognostic value in small-volume tumors^[17]^. Similarly, the SHAPE trial omitted LVSI assessment without apparent detriment to outcomes^[14]^. Emerging evidence suggests that focal LVSI (single focus) may not significantly worsen prognosis compared to absent LVSI, whereas diffuse LVSI (multiple foci) correlates with reduced survival^[23]^. While SEER data limitations precluded direct analysis of LVSI extent, our subgroup analyses revealed consistent survival across most cohorts except high-risk IB1 subgroups (adenocarcinoma/adenosquamous histology, tumors 11–20 mm, age >49 years). These observations tentatively suggest that tumor size and histology might outweigh LVSI status when combined with rigorous staging criteria, though further research is needed to confirm this hypothesis.

Regarding surgical approach, our study included both laparoscopic and open procedures without differentiation, a potential limitation given the LACC trial’s findings on minimally invasive radical hysterectomy^[24]^. However, exploratory analyses from the SHAPE trial found no significant differences in recurrence-free or overall survival between minimally invasive and open TH for low-risk disease^[25]^. Notably, residual disease rates were lower in minimally invasive cohorts (43.1% vs. 57.9%, *P* = 0.04) in the SHAPE trial, possibly reflecting selection bias toward smaller tumors. While our retrospective design precludes definitive conclusions about surgical approach, these data provide preliminary reassurance regarding technique variability in low-risk populations.

Strengths of this study include the large sample size from a population-based database, long follow-up period, and rigorous statistical methods including propensity score matching and multivariable adjustment. The study provides real-world evidence on the comparative effectiveness of total versus radical hysterectomy in early-stage cervical cancer. Several limitations warrant consideration. First, the retrospective design may introduce selection bias, as patients undergoing TH were likely perceived as lower-risk preoperatively. Second, our US-based population limits generalizability to regions with differing histologic profiles or screening practices; caution is advised when extrapolating these findings globally. Third, while we excluded patients with intraoperative lymph node involvement, variability in SLN mapping protocols across institutions may have influenced outcomes. Fourth, our risk stratification excluded socioeconomic variables such as marital status, despite their documented association with prognosis. While separated, divorced, or widowed patients exhibited poorer outcomes, these factors were deemed beyond the scope of biologically driven surgical decision-making in this study. Ongoing prospective trials will help address these uncertainties; however, large retrospective studies remain critical given the challenges of conducting randomized trials in low-risk populations and resource-limited settings. Future research should prioritize quality-of-life comparisons between conservative and radical approaches to better inform patient-centered decision-making.

## Conclusions

For FIGO IA2-IB1 cervical cancer patients without preoperative cone biopsy, TH may be appropriate for squamous/ adenocarcinoma/ adenosquamous carcinomas ≤2 cm with no radiographic extra-cervical spread. However, RH remains recommended for high-risk subgroups: IB1 adenosquamous/adenocarcinoma patients aged >49 years with 11–20 mm tumors. Our criteria omit LVSI and stromal depth assessment, simplifying eligibility without compromising safety, which is particularly valuable in resource-limited settings. These findings align with global efforts to expand access to conservative surgery.

## Data Availability

Readers can access the data used in this study from the links to public domain resources provided in the manuscript and Supplementary materials.

## Research registration unique identifying number (UIN)

This study is a retrospective study, and the data utilized have been sourced exclusively from publicly accessible databases. As such, the information presented does not necessitate Research Registration Unique Identifying Number.
